# Pathomechanisms of a *CLCN1* Mutation Found in a Russian Family Suffering From Becker's Myotonia

**DOI:** 10.3389/fneur.2020.01019

**Published:** 2020-09-04

**Authors:** Concetta Altamura, Evgeniya A. Ivanova, Paola Imbrici, Elena Conte, Giulia Maria Camerino, Elena L. Dadali, Alexander V. Polyakov, Sergei Aleksandrovich Kurbatov, Francesco Girolamo, Maria Rosaria Carratù, Jean-François Desaphy

**Affiliations:** ^1^Section of Pharmacology, Department of Biomedical Sciences and Human Oncology, School of Medicine, University of Bari Aldo Moro, Bari, Italy; ^2^N.P. Bochkov's Research Centre for Medical Genetics, Federal State Budgetary Scientific Institution, Moscow, Russia; ^3^Section of Pharmacology, Department of Pharmacy-Drug Sciences, University of Bari Aldo Moro, Bari, Italy; ^4^Voronezh Regional Clinical Consulting and Diagnostic Center, Voronezh, Russia; ^5^Unit of Human Anatomy and Histology, Department of Basic Medical Sciences, Neuroscience, and Sense Organs, School of Medicine, University of Bari Aldo Moro, Bari, Italy

**Keywords:** myotonia congenita, ClC-1, chloride channel, patch-clamp, intracellular trafficking

## Abstract

**Objective:** Myotonia congenita (MC) is a rare muscle disease characterized by sarcolemma over-excitability inducing skeletal muscle stiffness. It can be inherited either as an autosomal dominant (Thomsen's disease) or an autosomal recessive (Becker's disease) trait. Both types are caused by loss-of-function mutations in the *CLCN1* gene, encoding for ClC-1 chloride channel. We found a ClC-1 mutation, p.G411C, identified in Russian patients who suffered from a severe form of Becker's disease. The purpose of this study was to provide a solid correlation between G411C dysfunction and clinical symptoms in the affected patient.

**Methods:** We provide clinical and genetic information of the proband kindred. Functional studies include patch-clamp electrophysiology, biotinylation assay, western blot analysis, and confocal imaging of G411C and wild-type ClC-1 channels expressed in HEK293T cells.

**Results:** The G411C mutation dramatically abolished chloride currents in transfected HEK cells. Biochemical experiments revealed that the majority of G411C mutant channels did not reach the plasma membrane but remained trapped in the cytoplasm. Treatment with the proteasome inhibitor MG132 reduced the degradation rate of G411C mutant channels, leading to their expression at the plasma membrane. However, despite an increase in cell surface expression, no significant chloride current was recorded in the G411C-transfected cell treated with MG132, suggesting that this mutation produces non-functional ClC-1 chloride channels.

**Conclusion:** These results suggest that the molecular pathophysiology of G411C is linked to a reduced plasma membrane expression and biophysical dysfunction of mutant channels, likely due to a misfolding defect. Chloride current abolition confirms that the mutation is responsible for the clinical phenotype.

## Introduction

A difficulty in muscle relaxation after a voluntary contraction is the basis of the myotonic phenomenon, which is the main clinical feature of non-dystrophic myotonias (NDM). These disorders are caused by a dysfunction of skeletal muscle voltage-gated ion channels (1, 2). Thus, sodium channel myotonia and paramyotonia congenita are both linked to gain-of-function mutations of the *SCN4A* gene coding for Nav1.4 sodium channel, while myotonia congenita (MC) is related to loss-of-function mutations in the *CLCN1* gene, encoding the chloride channel ClC-1. MC can be inherited in a recessive mode (Becker's disease) or dominant manner (Thomsen's disease). Clinically, the two forms of MC differ by the age of onset, spreading of myotonia, and a typical transient muscular weakness present only in the recessive trait. Becker's disease is more common and generally more severe (3).

The ClC-1 chloride channel is expressed almost exclusively in skeletal muscle, where it accounts for ~80% of plasma membrane ion conductance at rest. The chloride current stabilizes the resting membrane potential of skeletal muscle and contributes to the repolarization of action potentials (4, 5). Thus, the reduced chloride conductance resulting from MC mutations predisposes the sarcolemma to spontaneous action potential runs or abnormal after-discharges that hamper muscle relaxation after contraction, causing myotonia.

So far, more than 200 mutations in *CLCN1* have been identified (5, 6) in patients with MC and a number of these have been functionally studied to confirm the genotype-phenotype relationship and to better understand the relationship between ClC-1 channel structure and function.

*In vitro* functional studies have demonstrated that MC mutations cause various alterations of channel function, including shift of voltage dependence, reduced single channel conductance, altered ion selectivity, or a defect in protein trafficking (5, 6). All these alterations reduce the activity of ClC-1 channel mutants, leading to a reduced sarcolemmal chloride conductance.

In this study, we report a mutation, p.G411C, identified in a Russian family affected by recessive MC. We have investigated the molecular defect of G411C chloride channels by means of a combined biochemical and electrophysiological approach in transiently transfected mammalian cells, in order to define the molecular mechanisms causing MC and to correlate it with the clinical manifestations of the affected patients.

## Materials and Methods

### Genetic Analysis

Written informed consent for DNA storage and use for genetic analysis and research purposes was obtained from all the patients and relatives, in accordance with the Declaration of Helsinki. Genomic DNA was extracted from peripheral blood cells according to the standard method with DLAtom™ DNA Prep100 kit. All the 23 exons of *CLCN1* were amplified by polymerase chain reaction (PCR) and were sequenced using intronic primers, as previously described (7).

### Clinical Diagnosis

We examined a young Russian boy presenting with muscle stiffness and his relatives. Neurological examination was specifically conducted to search for myotonic signs as tongue, eyelid, lid-lag, jaw, handgrip, and percussion myotonia. EMG study was performed according to Fournier's guidelines (8).

### Mutagenesis and Expression of WT and G411C Mutant hClC-1 Channels

The c.1231G>T mutation was introduced into the plasmid pRc/CMV containing the full-length WT hClC-1 cDNA using the QuickChange™ site-directed mutagenesis kit (Stratagene Cloning Systems), as previously described (9). For confocal imaging, point mutation was inserted into pRcCMV-YFP-hClC-1 vector, kindly provided by Dr. Christoph Fahlke (10).

The complete coding region of the cDNA was sequenced to exclude polymerase errors. Human embryonic kidney 293T (HEK293T) cells were transiently transfected with a mixture of wild-type or mutated hClC-1 (5 μg) and CD8 reporter plasmids (1 μg) using the calcium–phosphate precipitation method. The transfected cells were maintained at 37°C for 40–80 h before being used for electrophysiological or biochemical experiments. Where indicated, drugs (MG132, dithiothreitol) were diluted in the culture medium.

### Electrophysiology and Data Analysis

Transfected HEK293T cells were examined between 40 and 80 h after transfection. Only cells decorated with anti-CD8 antibody-coated microbeads (Dynabeads M450; Invitrogen, Carlsbad, California, USA) were used for electrophysiological studies.

Standard whole-cell patch-clamp recordings were performed at room temperature (~20°C) using an Axopatch 200B amplifier and pClamp suite software (Axon Instruments), as previously described (11, 12). The composition of the extracellular solution was (in mM): 140 NaCl, 4 KCl, 2 CaCl_2_, 1 MgCl_2_ and 5 Hepes, and the pH was adjusted to 7.4 with NaOH. The pipette solution contained (in mM): 130 CsCl, 2 MgCl_2_, 5 EGTA and 10 Hepes, and the pH was adjusted to 7.4 with CsOH. In this condition, the equilibrium potential for chloride ions was about −2.8 mV and cells were clamped at the holding potential (HP) of 0 mV. Pipettes were pulled from borosilicate glass and had <3 MΩ resistance, when filled with the above pipette solutions. Currents were low-pass filtered at 2 kHz and digitized with sampling rates of 50 kHz using the Digidata 1440A AD/DA converter (Axon Instruments). Chloride currents were recorded ~5 min after achieving the whole-cell configuration, to allow the pipette solution to diffuse into the cell. Patches with a series resistance voltage error >5 mV and those with non-negligible leak current were discarded.

We measured the I–V relationship and the overall apparent open probability in high-chloride (134 mM) intracellular solutions to enhance current amplitude. The holding potential (HP) was set at 0 mV. Voltage steps of 400 ms were applied from −150 to +150 mV in 10 mV intervals, each followed by a voltage step at −105 mV to record tail currents. Voltage steps were applied every 3 s to allow complete recovery of current amplitude at the HP between two pulses. Data were analyzed off-line by using pClamp 10.3 (Axon Instruments) and SigmaPlot 8.02 (Systat Software GmbH) software. The instantaneous and steady-state current amplitudes were measured at the beginning (~1 ms) and end (~390 ms) of each voltage step and normalized by cell capacitance to calculate current densities in pA/pF. Current densities are reported as means ± S.E.M from n cells and statistical analysis was performed using Student's *t*-test, with *P* < 0.05 considered as significant.

For pharmacological experiments, transfected HEK293T cells were incubated with the proteasome inhibitor MG132 (MG-132 Ready Made Solution, Sigma-Aldrich, M7449) at the final concentration of 20 μM for 16 h and then used for patch-clamp recordings. The reducing agent dithiothreitol (DTT, Pierce, Rockford, IL, USA) was added at the final concentration of 1 or 2 mM in the culture medium for cell incubation or in the extracellular patch-clamp solution for acute gravity-driven diffusion around the patched cell (13).

### Biotinylation of Cell-Surface Proteins

Cell-surface biotinylation was carried out with the Pierce Cell Surface Protein Isolation Kit (Pierce, Rockford, IL, USA). Transfected HEK293 cells were incubated for 16 h at 37°C in the absence or presence of 20 μM MG132 and, subsequently, cell-surface proteins were labeled with sulfosuccinimidyl-2-(biotinamido) ethyl-1,3-dithiopropionate (Sulfo-NHS-SS-biotin). In brief, cells were washed twice with ice-cold PBS and incubated with Sulfo-NHS-SS-biotin in PBS for 30 min at 4°C, with gentle rocking on an orbital shaker. Excess biotin was quenched with quenching solution. Cells were treated with lysis buffer and centrifuged at 10,000 *g* for 2 min at 4°C. Clear supernatant was reacted with immobilized NeutrAvidin gel slurry in columns for 60 min at room temperature. After centrifugation, cytoplasmic proteins were recovered from the flow-through, whereas surface proteins were obtained after elution with a sample buffer containing DTT. Both samples were quantified by using Pierce™ BCA Protein Assay Kit—Reducing Agent Compatible (Thermo Scientific, USA) and then used for Western Blot experiments.

### Western Blot Analysis

To measure total ClC-1 protein expression, HEK293 cells were transfected with pRcCMV-hClC-1 WT or G411C constructs (5 μg each) and then incubated for 16 h in the absence or presence of 20 μM MG132. After incubation, cells were harvested in 200 μl of cold RIPA buffer (20 mM Tris-HCl, 150 mM NaCl, 1,5% Non-idet P-40, 100 mM sodium orthovanadate, 10 mg/ml PMSF and a protease inhibitor cocktail) and placed for 10 min in ice. To complete cell lysis, suspensions were passed through a syringe with a needle for 10 times. After 15 min in ice, cell lysates were centrifuged at 14,000 rpm for 30 min at 4°C, and supernatant was collected. Total protein amounts were quantified by using a BCA protein assay kit (Bio-Rad, Hercules, CA, USA).

Total proteins or surface and cytoplasmic proteins from biotinylation assay (8 μg) were separated on a 10% SDS-PAGE and transferred onto nitrocellulose membrane for 1 h at 200 mA (SemiDry transferblot, Bio-Rad). Membrane was blocked for 2 h with 0.2 M Tris-HCl, 1.5 M NaCl, and pH 7.4 buffer (TBS) containing 5% non-fat dry milk and 0.5% Tween-20 and incubated overnight at 4°C with rabbit anti-ClC-1 antibody–C-terminal (ab189857, Abcam) diluted 1:500 and monoclonal mouse anti-Actin (Sc-47778, Santa Cruz Biotechnology) diluted 1:300 with TBS containing 5% non-fat dry milk. After three washes with TBS containing 0.5% Tween-20 (TTBS), membrane was incubated for 1 h with goat anti-rabbit IgG conjugated to horseradish peroxidase (Biorad). Membrane was then washed with TTBS, developed with a chemiluminescent substrate (Clarity Western ECL Substrate; Bio-Rad), and visualized on a Chemidoc imaging system (Bio-Rad). Western blots were quantified with Image Lab software (Bio-Rad), which allows the chemiluminescence detection of each experimental protein band to obtain the absolute signal intensity automatically adjusted by subtracting the local background. For total protein expression, density was standardized as the ratio of the ClC-1 signal to the cognate β-actin signal. For biotinylation assay, the distribution of ClC-1 was quantified by calculating the ratio of surface proteins to the sum of surface and cytoplasmic proteins. Quantitative analysis was performed from 3 to 4 independent experiments. Statistical analysis was performed using ANOVA followed by Sidak's multiple comparisons test (Prism 8.4.3, GraphPad Software Inc.).

### Confocal Imaging

The HEK293T cells were seeded on 1X polylysine-treated culture dishes (CELLview™–Cell Culture Dish with Glass Bottom 627860, Greiner) and then transfected with cDNA encoding wild-type or G411C YFP-hClC-1 (1 μg) using the calcium-phosphate precipitation method.

Two days after transfection, cell plasma membrane was stained with wheat germ Agglutinin, Alexa Fluor™555 Conjugate (WGA-555W32464, Thermo Fisher Scientific, Waltham, MA, USA) in PBS for 10 min at 37°C at a final concentration of 3 μg/ml. Confocal images were obtained using a Leica TCS SP5 confocal laser scanning microscope (Leica Microsystems, Mannheim, Germany). The YFP-ClC-1 fluorescence was excited with a green argon laser (450–500 nm of excitation wavelength) and recorded at 490–550 nm. The WGA-555 fluorescence was excited using red HeNe 543 laser, selecting an excitation at 500–590 nm and emission at 550–650 nm. Single confocal optical planes, containing a cluster of one to five transfected cells, were collected with a 63x oil lenses, using a sequential scan procedure at 0.45 μm intervals through the z-axis of the section.

Quantitative evaluation of the colocalization of YFP-ClC-1 and WGA-555 fluorescence pixels was performed by two independent observers (AC and FG), blinded for group allocation, using ImageJ JACoP plugin (NIH, Bethesda, MD, USA), according to developer's instructions (14). Analysis was performed on 2–4 optical planes (2.25 μm intervals) of 64 WT and 34 GC different fields containing 1–5 cells. A total of 101 WT and 50 GC cells were thus analyzed. The selected fields were segmented using the automatic threshold calculated by JACoP for the two channels (red and green); the resulting pairs of binary images were then analyzed to obtain the Pearson's correlation coefficient (*P*) and the Mander's colocalization coefficients (M1 and M2). The pixel intensity scatter-plots were obtained by using EzColocalization plugin for ImageJ according to developer's instructions (15). The results were expressed as mean value ± *SD*. Statistical analysis was performed using unpaired Student's *t*-test. Differences were considered significant with *P* < 0.05.

## Results

### Clinical Report

**Figure 1:**
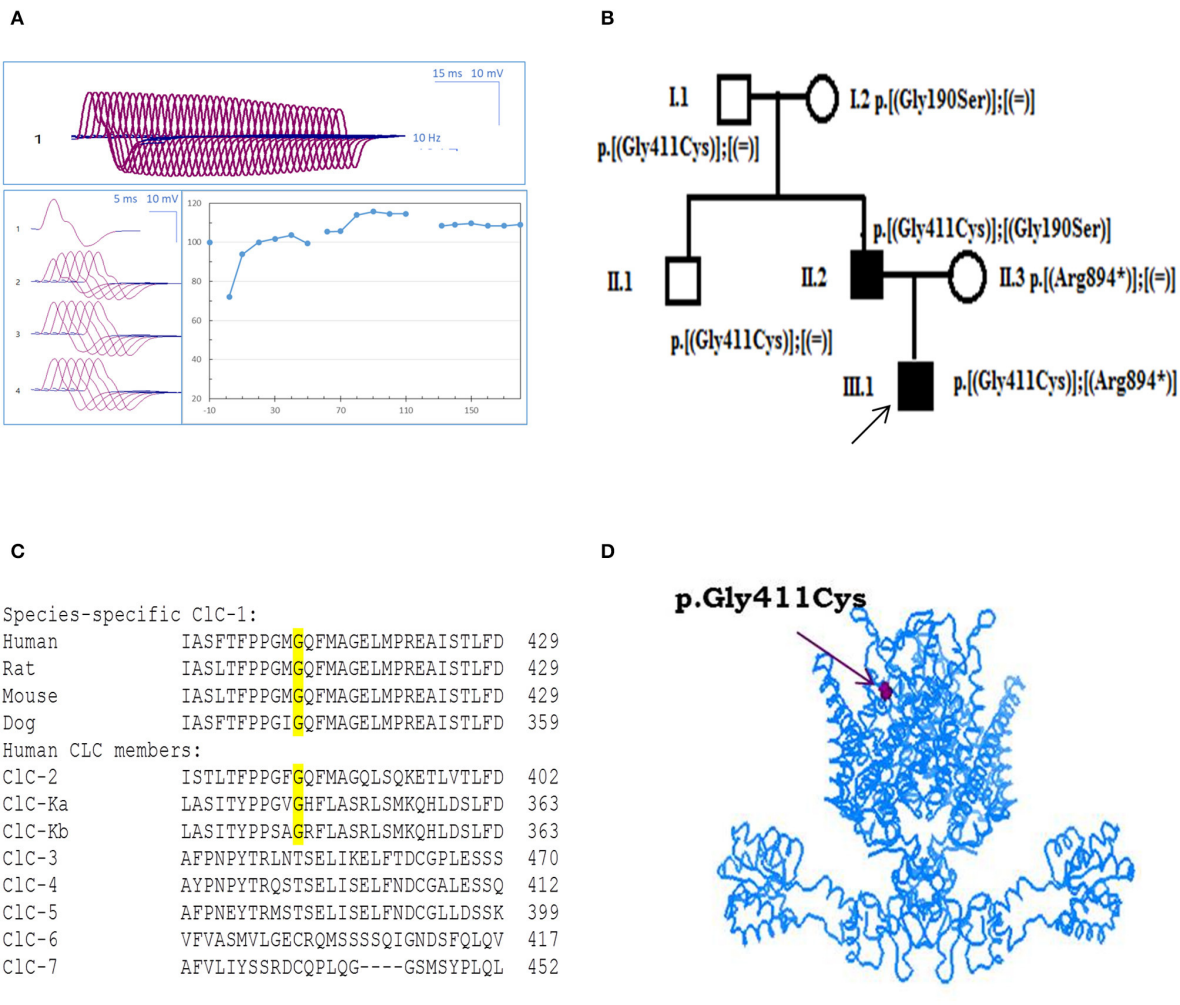
Probands' EMG, pedigree, and ClC-1 mutation. **(A)** Repetitive nerve stimulation (RNS) on *abductor digiti minimi* muscle. **(B)** Pedigrees of the Russian family affected by Becker disease. Squared symbols indicate men and round symbols indicate women. Empty symbols denote healthy individuals and dark filled symbols denote affected individuals. The arrow indicates the proband in the family. **(C)** Amino acid alignment of the ClC-1 chloride channel among different species and human CLC members. **(D)** Three-dimensional representation of hClC-1 channel modeled upon the X-ray structure of a eukaryotic Cl^−^/H^+^ exchanger CmClC showing the localization of G411C mutation.

The proband showed generalized muscle stiffness for the first time at the age of 1 year, when he begun to walk on his own. Clinical examination was carried out at the age of 5 years and revealed muscle hypertrophy and percussion myotonia with warm up phenomenon. Electromyography (EMG) study in proximal and distal muscles confirmed electrical myotonia and showed a 24.5% decrement of the amplitude of the compound muscle action potential at repetitive 10-Hz nerve stimulation (Figure 1A). The short exercise test revealed characteristic EMG pattern II generally associated with defects in the chloride channel (16). The tendon reflexes on hands and legs were decreased, whereas muscle force in proximal parts of hands and legs was normal without muscle weakness. Probands' father showed myotonic signs similar to his son and was treated with acetazolamide 500 mg/day with no improvement.

The c.1231G>T (p.Gly411Cys) recessive mutation in *CLCN1* was detected in the proband in compound heterozygosis with the already known c.2680C>T mutation (p.Arg894^*^) (17). Probands' father also carried p.Gly411Cys mutation, in association with the well-known c.568GG>TC transition (p.Gly190Ser) (9, 18, 19). Conversely, the relatives who carried G411C, R894X, or G190S mutation in heterozygosis with WT showed no sign of myotonia, confirming a recessive inheritance of the disease (Figure 1B).

### Functional and Biochemical Characterization of G411C Mutant Channels

Using Clustal Omega, multiple amino acid sequence alignment showed that G411 residue is well-conserved among ClC-1 of mammals and various human CLC proteins (ClC-2 and ClC-K) (Figure 1C). Positioning of the mutation in the 3D structural model of hClC-1 channel modeled upon the structure of CmClC (PDB id: 3ORG) (20, 21) suggested that G411 is located in the transmembrane region, between the α-helix domains K and L (Figure 1D), far away from the conducting pore of the channel (22–24). Analysis using MutPred software scored G411C substitution with 0.8 probability to be deleterious.

To understand better the consequence of the mutation and its role in MC, we performed functional characterization of G411C ClC-1 channels using the patch-clamp technique.

**Figure 2:**
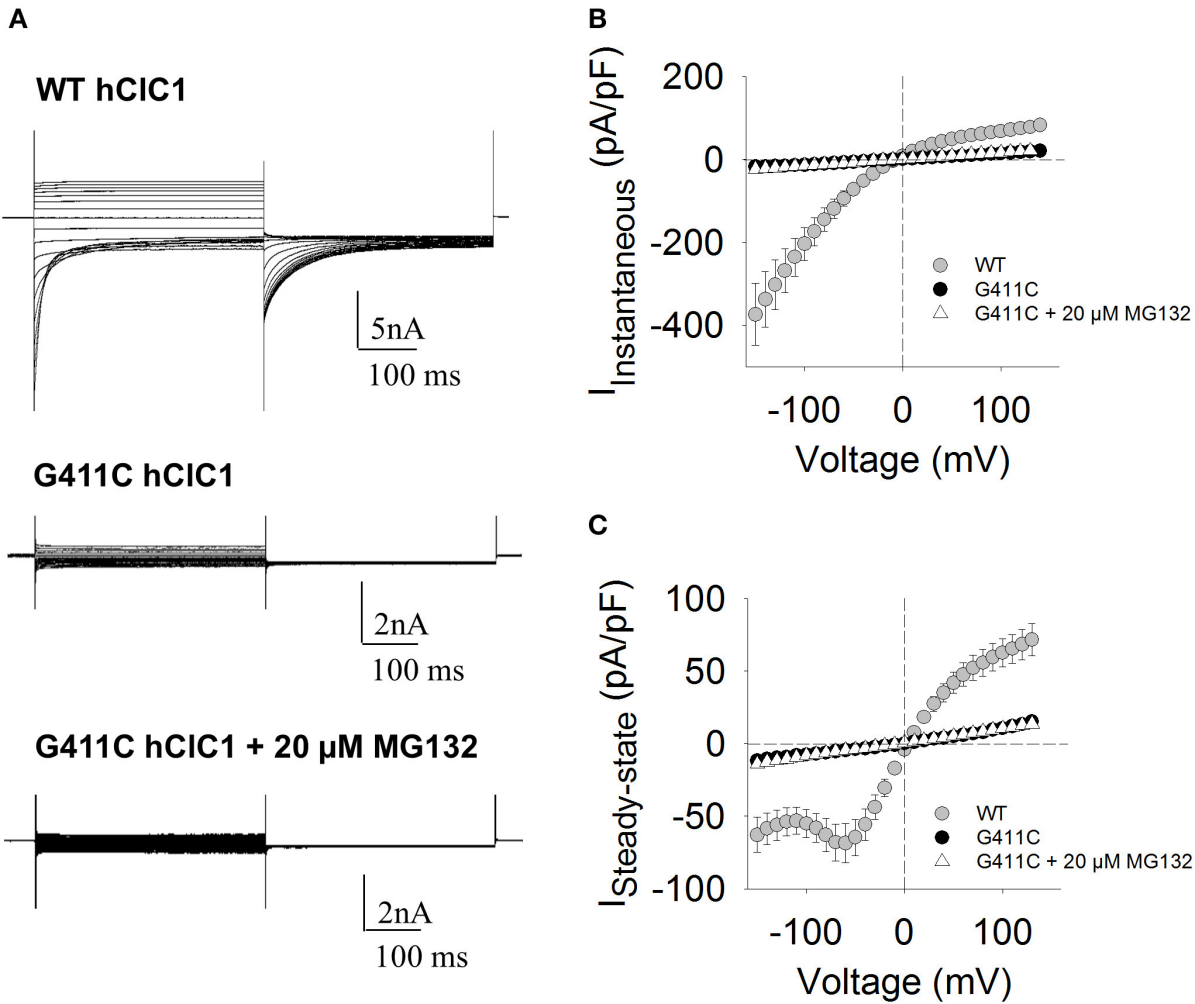
Chloride currents of WT and G411C hClC-1 channels expressed in HEK293T cells. **(A)** Representative chloride currents recorded from HEK293T cells transfected with 5 μg WT or G411C hClC-1 cDNAs in high-chloride intracellular solution. The G411C currents were also measured after 16 h incubation with 20 μM MG132. The holding potential (HP) was set at 0 mV. Voltage steps of 400 ms were applied from −150 to +150 mV in 10 mV intervals, each followed by a voltage step at −105 mV to record tail currents. Voltage steps were applied every 3 s to allow complete recovery of current amplitude at the HP between two pulses. **(B)** Instantaneous currents were measured at the beginning (~1 ms) of voltage step and normalized with respect to cell capacitance (pA/pF) to calculate current density. **(C)** Steady-state current densities were measured at the end of voltage step (~390 ms). Each data point is the mean ± S.E.M. from 10 to 12 cells.

For WT channels, instantaneous currents were observed at each voltage steps, which decreased over time between −150 and −60 mV (corresponding to current deactivation) or remained stable within 400 ms between −60 and +150 mV. Current amplitudes saturated at voltages >+50 mV. In contrast, transfected HEK293T with ClC-1 mutant did not generate any chloride current within the voltage range of −150/+150 mV (Figure 2).

**Figure 3:**
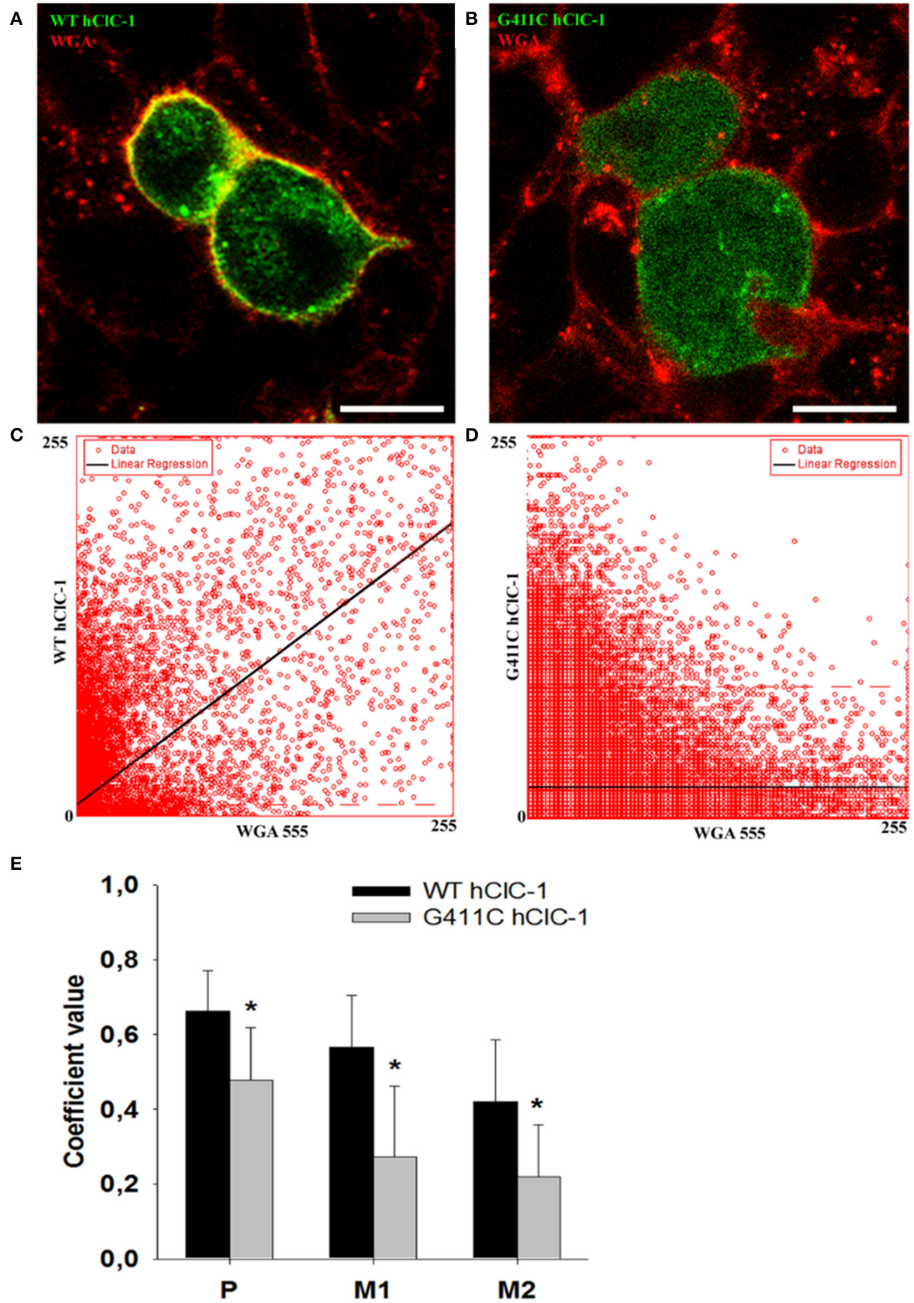
Confocal microscopy images of HEK293T cells expressing wild-type YFP-ClC-1 channel and YFP-ClC-1 G411C mutant. **(A)** Representative confocal single optical plane image showing localization of wild-type YFP-ClC-1 (green signal), plasma membrane marker WGA-555 (red signal), and colocalization (yellow signal) in a dividing cell doublet. **(B)** Representative dividing cell doublet showing localization of YFP-G411C (green signal), plasmalemma WGA-555 (red signal), and colocalization (yellow signal). **(C,D)** Scatter-plots of pixel intensity in images **(A,B)**, showing the relationship between WT or G411C and WGA. Linear regressions (black lines) demonstrate significant colocalization of WT hClC-1 with WGA [Pearson's correlation coefficient (*P*) of 0.7] and anticolocalization of G411C with WGA (*P* = 0.4). **(E)** Histograms reporting the Pearson's coefficient (*P*) of pixel intensity scatter-plot linear regressions (as in **C,D**), and the Mander's overlap coefficients (M1 and M2) of the two markers (WGA-555 and YFP-ClC-1). M1 indicates summed intensities of red pixels overlapping with green to the total red intensity (proportion of WGA overlapping with ClC-1). M2 indicates summed intensities of green pixels overlapping with red to the total green intensity (proportion of ClC-1 overlapping with WGA). Each bar is the mean ± *SD* from 64 (WT) and 34 (G411C) cell clusters. *p < 0.001 between WT and G411C hClC-1 with unpaired Student's *t*-test.

To verify whether G411C channels were expressed or not, we constructed fluorescent plasmids containing cDNA encoding YFP-tagged WT or mutant ClC-1 proteins, and we examined transfected cells using confocal imaging (Figure 3). We used wheat germ agglutinin AlexaFluor™555 to stain cell plasma membrane and evaluated the co-localization with the YFP-ClC-1. Wild type ClC-1 (green) was mainly expressed at the plasma membrane level as shown by the high degree of co-localization with WGA-555 (Figure 3). Conversely, the G411C hClC-1-YFP protein was little present on the cell surface and green fluorescence was essentially cytoplasmic, suggesting that the mutation induced a defect in subcellular distribution.

**Figure 4:**
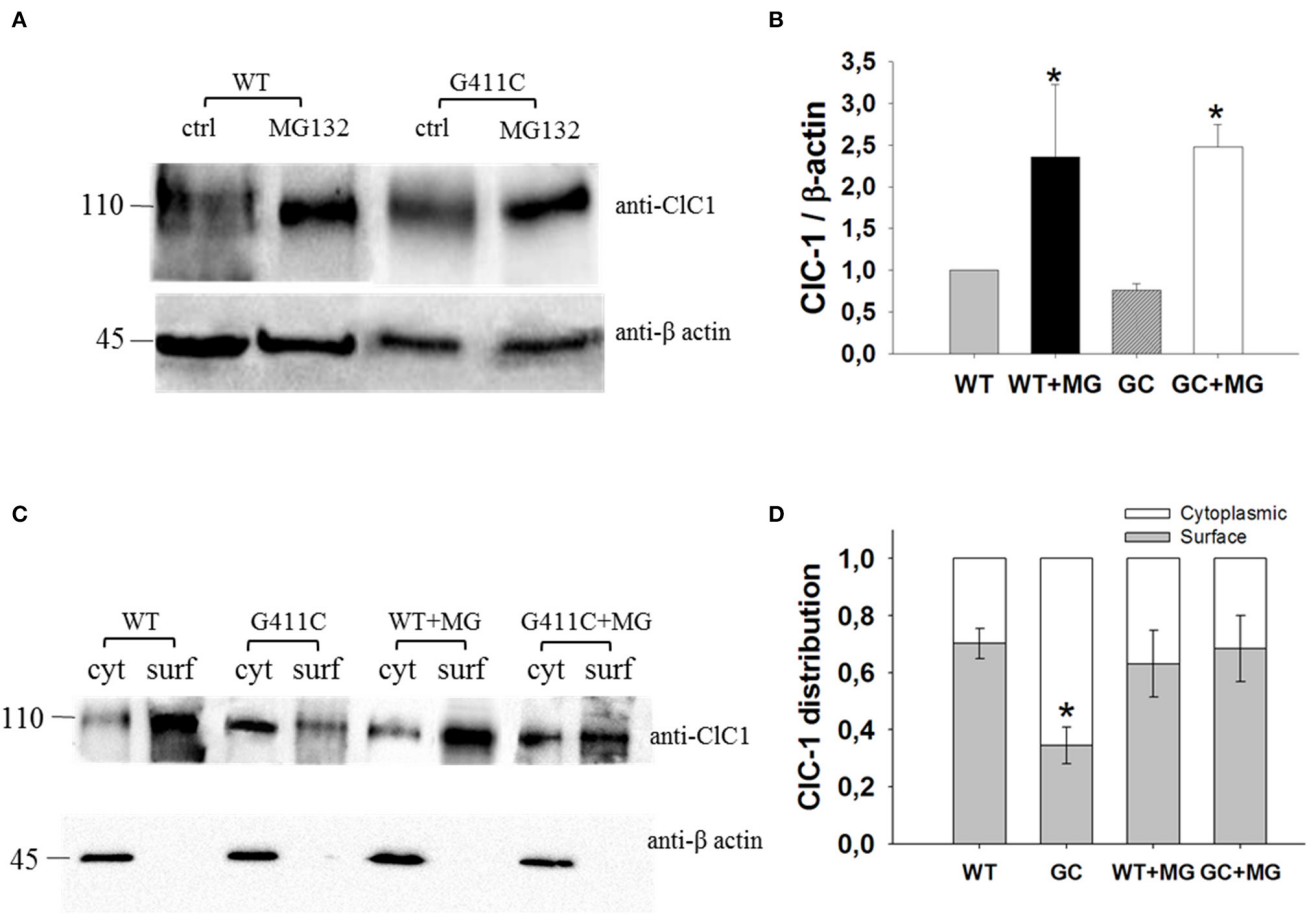
ClC-1 protein expression in HEK293T cells transfected with WT or G411C ClC-1 channels and effects of MG132 incubation. **(A)** Representative western blot of total ClC-1 and β-actin proteins from transfected HEK293T incubated for 16 h in absence or presence of 20 μM MG132. **(B)** Quantification of ClC-1 total protein expression level. The ClC-1 signal of each column was standardized to β-actin signal and normalized to WT on the same blot. Each bar is the mean ± S.E.M. from three independent experiments. Asterisks denote significant difference (*p* < 0.04) between control condition and MG132 treatment using two-way ANOVA. **(C)** Representative western blot of surface and cytoplasmic ClC-1 and β-actin proteins obtained from biotinylation assay of transfected HEK293T cells incubated for 16 h in absence or presence of 20 μM MG132. **(D)** Quantification of surface and cytoplasmic ClC-1 protein distribution. Each bar is the mean ± S.E.M. from 3 to 4 independent experiments. Statistical analysis was performed using two-way ANOVA followed by Sidak's multiple comparison test (* at least *p* < 0.05 vs. WT and GC + MG132).

We thus investigated total ClC-1 protein expression level and ClC-1 distribution within cell compartments using Western Blotting analysis and biotinylation assay. Quantitative analysis revealed no significant difference between WT and G411C in total protein expression level (Figures 4A,B). Noticeably, the distribution of G411C protein between plasma membrane and cytoplasmic compartments was inverted compared to WT (Figures 4C,D), with a dramatically reduced surface expression of the mutant (−51% compared to WT), in full agreement with confocal imaging analysis.

### Effect of the Proteasome Inhibitor MG132 on WT and Mutant hClC-1

Several mechanisms might have been responsible for the dramatic reduction of G411C protein surface expression, including an increase in protein degradation. We explored the possibility that G411C mutation could induce a folding defect in ClC-1 protein, making it more sensitive to the ubiquitin-proteasome system. For these experiments, we exploited peptide aldehydes such as MG132, commonly used to examine the involvement of this mechanism.

Transfected HEK293T were incubated with 20 μM of MG132 for 16 h and then used for Western Blot analysis of total protein or surface and cytoplasmic proteins (biotinylation assay). MG132 treatment significantly increased the total protein level of WT and G411C mutant by more than 2-fold (Figures 4A,B). No significant difference was observed in wild-type ClC-1 protein distribution within plasma membrane and cytoplasm. However, MG132 treatment enhanced G411C protein expression at the plasma membrane, showing a subcellular distribution more similar to WT (Figures 4C,D).

We next verified if the MG132 treatment also restored chloride currents. Figure 2 shows the effect of MG132 treatment on G411C mutant channel, after 16 h of incubation. Surprisingly, despite the increase of the surface protein level as evidenced by biotinylation assay, no significant chloride current was recorded from the G411C-transfected cell treated with MG132, suggesting that this mutation produced non-functional ClC-1 chloride channels.

### Effect of the Reducing Agent DDT on G411C hClC-1

Because the mutation introduces a cysteine residue in ClC-1, we wondered whether the formation of unnatural disulfide bridge might account for channel defect. In a series of experiments, the HEK cells transfected with G411C were exposed to the reducing agent DTT (1 or 2 mM) either acutely during patch-clamp recordings or through cell incubation for up to 2 h. According to the crystal structure, G411C is located close to the external surface of the protein and should be thus easily accessible to DTT. In no case, DTT was able to restore G411C chloride currents, suggesting that the cysteine impairs channel function without forming disulfide bond (not shown).

## Discussion

In the present manuscript, we reported a detailed study of a ClC-1 mutation, p.G411C, identified in a Russian family. This mutation was detected only recently in a large group of patients with skeletal muscle channelopathies from the Netherlands (25). The mutation was associated with the frameshift mutation Phe404Hisfs^*^16, suggesting a recessive trait, but was not functionally characterized. The Russian pedigree confirms such a recessive inheritance, as the probands' grandfather and uncle carrying G411C in heterozygosis with WT were asymptomatic. In the symptomatic proband and his father, the mutation was associated in compound heterozygosis with two well-known mutations, p.R894X (17) and p.G190S (9), respectively. Thus, the inheritance pattern and the clinical examination of both patients suggested a Becker's phenotype associated with severe myotonia.

The pathogenicity of p.G411C was further confirmed by functional and biochemical studies. In transfected HEK293T cells, p.G411C mutant did not produce discernible chloride currents, which can account for muscle hyperexcitability and myotonic discharges. The associated p.R894X mutation deletes 94 amino acids of the C-terminus of the protein, leading to a large reduction of chloride conductance due to a decrease of surface protein expression (17, 26). The p.G190S mutation, identified in compound heterozygosis in the father, induces a dramatic shift of the open probability voltage dependence toward very positive voltages, resulting in nearly zero chloride current within the physiological range of sarcolemma voltage (9). The coexistence of p.G411C with these mutations may result in a huge reduction of the sarcolemma chloride conductance and is therefore likely responsible for the severe myotonic phenotype.

Biochemical studies suggest that p.G411C does not impair total protein expression, but rather reduces the fraction of the protein expressed at the surface membrane. Impaired subcellular distribution of G411C mutant channel was also confirmed by confocal imaging studies.

Because of the evident recessive inheritance mode of p.G411C, a dominant-negative effect on wild-type is unlikely. This suggests that either G411C does not significantly assemble with WT to form heterodimers or WT-G411C heterodimers can reach the membrane and WT protopore work normally. In both cases, WT channels would ensure at least 50% of the sarcolemma chloride conductance, which is sufficient to guarantee normal muscle function.

Interestingly, several myotonia-causing mutations are located near G411 residue, such as p.P408A (27), p.Q412P (28), p.F413C (26, 29, 30), p.A415V (31), pG416E (32), and p.E417G (33). Among these, p.Q412P was shown to induce a drastic reduction of ClC-1 chloride currents in *Xenopus* oocytes and HEK293 cells, as the consequence of a severe folding defect, rendering ClC-1 protein more susceptible to its degradation (28). Yet, the small proportion of mutated channels reaching the plasma membrane showed biophysical properties undistinguishable from wild-type. The F413C mutant was shown to impair channel trafficking in transfected myotubes (26) and to induce small changes in chloride current voltage–dependence and kinetics in transfected HEK293 cells (30). Similar to G411C, G416E did not produce any chloride current in transfected HEK293 cells (32). Thus, mutations located in the K-L loop may have different effects on chloride channel gating but all impair the ClC-1 channel surface expression, suggesting an important role of the loop in channel trafficking. It is also worth noting that all these mutations are recessive.

Post-translational alteration of ion channel expression can occur at different levels: altered intracellular trafficking, increased turnover at the plasma membrane, or increased degradation rate (34, 35). Misfolded proteins may be retained into the ER and redirected through the ER-associated degradation (ERAD) pathway toward proteasomal degradation. Misfolded proteins reaching the plasma membrane may also be removed by the peripheral quality control system and degraded through the endosome/lysosome pathway. Such mechanisms are also valid for misfolded ClC-1 mutants causing myotonia congenita (7). In the case of G411C, we observed that the total protein expression level was similar to WT, suggesting that there was no enhancement of protein degradation. Rather, the altered distribution of G411C between surface and cytoplasm suggests a trafficking defect of the mutant toward the plasma membrane and/or an increased turnover at the plasma membrane. We incubated transfected HEK cells with MG132 in order to assess the role of the proteasome in G411C distribution (36). As expected, MG132 treatment increased total G411C protein expression in a manner similar to WT, arguing for a normal degradation rate of the mutated protein. Importantly, the treatment with MG132 also restored the cellular distribution of the G411C mutant. A possible explanation might be that inhibition of the proteasome enables G411C mutant to leave the ER and reach the plasma membrane more efficiently, maybe through modulation of molecular chaperone activity. Dedicated experiments would be needed to confirm such hypothesis. Despite an increase of the surface protein level of G411C by the MG132 treatment, no significant increase in chloride current was observed in patches recorded from the G411C-transfected cells. Taken together, these results suggest that G411C mutation disrupts the folding of ClC-1 protein, making the chloride channels non-functional and trafficking-defective.

In conclusion, the present study expands the spectrum of *CLCN1* mutations responsible for MC and contributes to the understanding of genotype-phenotype correlation. The improved understanding of the molecular mechanisms underlying MC could help the discovery of new drugs targeting specific mutant channels defects. The ideal pharmacological approach would point to the development of molecules able to correct biophysical defects in case of gating-defective mutations, as for G190S mutation, or able to restore protein surface expression, as for R894X and G411C mutants. Pharmacological chaperones could represent the elective tools able to stabilize protein correct folding and stability and to limit ER retention, thus overcoming the membrane expression defect and allowing the development of a personalized treatment for MC patients. Recently, two small molecules inhibiting the ubiquitination of ClC-1 in the ER proved effective in correcting the impaired biogenesis of misfolded ClC-1 protein (37, 38). However, in the case of G411C, the drug should be able, not only to restore cell surface expression, but also to improve channel function.

## Data Availability Statement

The datasets generated for this study can be found in the Leiden Open variation database (#00269797).

## Ethics Statement

The studies involving human participants were reviewed and approved by Ethics committee of the FSBI N.P. Bochkov's Research Centre for Medical Genetics, Moscow, Russia. Written informed consent to participate in this study was provided by the participants' legal guardian/next of kin.

## Author Contributions

CA: *in vitro* experiment data acquisition, analysis, interpretation, and manuscript writing. PI, EC, and GC: *in vitro* experiment data acquisition. FG: confocal imaging experiments and contribution to manuscript writing. EI: clinical, genetic data acquisition, and contribution to manuscript writing. ED, AP, and SK: clinical and genetic data acquisition. MC: critical revision of manuscript. J-FD: study concept, design, interpretation of data, study supervision, and critical revision of manuscript. All authors contributed to the article and approved the submitted version.

## Conflict of Interest

The authors declare that the research was conducted in the absence of any commercial or financial relationships that could be construed as a potential conflict of interest.
